# PD19 - Co-recognition of lipid transfer protein in pollen and foods in a Greek pediatric population

**DOI:** 10.1186/2045-7022-4-S1-P19

**Published:** 2014-02-28

**Authors:** Dimitrios Karantoumanis, Savvas Savvatianos, Anastasios P Konstantinopoulos, Dimitrios Koutsalitis, Anna Pananaki, Maria Psomiadou, Kassiani Tzeli, Marianna Tziotou, Dimitrios Mitsias, Eirini Roumpedaki, Stavroula Giavi, Paraskevi Xepapadaki, Nikolaos Douladiris, Emmanouil Manousakis, Nikolaos G Papadopoulos

**Affiliations:** 1 Allergy Department, 2nd Pediatric Clinic, University of Athens, Athens, Greece

## Background

Plant-food allergy is the food allergy most commonly found in older children and adults. Lipid transfer proteins (LTPs) are plant panallergens that are considered clinically relevant in plant-foods, especially in the Mediterranean area. An LTP syndrome, characterized by multiple, unstable reactivity to related plant-food allergy is not uncommon in the area. The peach LTP dominates the immunological response to these proteins but LTPs are present in pollens from several anemophylous plants and this has led to the suggestion that the primary sensitization to this allergen might occur through the airways, as a result of contact with one of these pollens.

## Objective

The present study looked at the prevalence of hypersensitivity to different LTP-containing pollen sources among allergic subjects with an LTP-syndrome.

## Methods

Twenty-three children (17 male; mean age 9.5 years) with LTP-syndrome living in Greece, underwent skin prick tests with commercial whole extracts (ALK-Abello) for peach, mugwort, plane and olive pollens.

## Results

Skin Tests with Peach, Artemisia, Platanus and Olea extracts scored positive (≥3mm) in 23 (100%), 15 (65%), 10 (43%) and 10 (43%) subjects, respectively.

## Conclusions

In our population mugwort, plane and olive pollen seem an unlikely source of primary LTP sensitization; the most likely primary sensitizer to this protein remains the peach (or a closely related plant-food), via the skin or the airways, in agreement with results from Northern Italy.

